# Announcement and Program Texas State Medical Association, Etc.

**Published:** 1893-04

**Authors:** 


### Society Notes.

### ANNOUNCEMENT AND PROGRAM OF THE TEXAS STATE MEDICAL ASSOCIATION; 25th ANNUAL CONVENTION, TO BE HELD AT GALVESTON, May 2nd, 3rd, 4th, and 5th, 1893.

*To the members of the State Association, and the Regular Medicat Profession in Texas:*

You are cordially invited to attend the forthcoming twenty-fifth annual session of the State Association, commencing Tuesday, May 2nd. If a member of the Association, you are urged to attend and contribute to its scientific work. If not a member, be present and identify yourself with the representative medical organization of the State.

H. A. WEST. M. D., Secretary.

---

PRESIDENT'S OFFICE, CLEBURNE, TEXAS, April 1, 1893.
*To the Members of the Texas State Medical Association:*

It is my duty and pleasure to remind you that the Twenty-Fifth Annual session of the State Medical Association will be held in Galveston from May 2nd to 5th inclusive. The city of Galveston, always a delightful one to visit, is especially attractive in the flowery month ot May. I am assured by the Chairman of the Committe of Arrangements that everything for the comfort, pleasure and convenience of those in attendance has been looked after. The social features of the occasion will include an entertainment at the Garten Verein and an excursion on Galveston Bay. The Secretary informs me that the list of papers promised so far insures an interesting and instructive program. The necessity of organization and co-operation of the medical profession was never more apparent than at this time, and in the State of Texas. We can never hope to accomplish the needful reforms in medical legislation except through the enlightenment of public opinion through the organized medical profession. A committee was appointed for the codification and revision of the Constitution and By-Laws, of which Dr. J. H. Sears of Waco, is Chairman; it is hoped that this committee will formulate some plan by which the various county and district societies may be brought into more intimate relations with the State Association. These and numerous other considerations impel me to urge a large attendance. Fraternally yours,

J. D. OSBORN, M. D., Pres't

REDUCED RAILROAD RATES.

Mr. H. G. Thompson, General Passenger and Ticket Agent of the Gulf Colorado & Santa Fe Railway, authorizes me to make the following statement:

GALVESTON, TEXAS, April 4, 1893.

*H. A. West, M. D., No. 2107 Market St., Galveston.*

DEAR SIR;--In response to your favor of March 18th and April 3rd, relative to delegates attending the State Medical Association at Galveston May 2nd. For the occasion the Gulf, Colorado & Santa Fe Railway will make a rate of one and one-third (1⅓) fares for the round trip on the certificate plan, and from advices received, other Texas lines will undoubtedly make the same concession. The certificate plan provides that where passengers pay tariff rates from starting point to Galveston, they will receive a receipt or certificate showing form, route and rate of ticket purchased, and this certificate when signed by the Secretary and countersigned by Joint Agent, will entitle the holder to purchase ruturn ticket at rate of one-third (⅓) fare, thus making rate of fare and one-third for the round trip. The Secretary of the Association should gather all certificates from delegates and present to the Joint Agent of the Texas lines, who in this case, will be Mr. G. B. Nichols, City Ticket Agent of the I. & G. N. R. R. When these certificates are signed by yourself as Secretary, and countersigned and stamped by Mr. Nichols as Joint Agent, they will be accepted by agents of the I. & G. N. R. R., and the G. C. & S. F. R'y at Galveston as authority for rate of one-third(⅓) fare returning, with certain limitations; the first of which is the going ticket must not have been purchased prior to May 1st, and that the return ticket must be purchased not later than one day from date of which meeting adjourns. Circulars will go to our agents instructing them in this matter, and advising them in regard to certificates for this occasion.

However, it would not be out place when issuing your notices to members of your Association to say to them, that on purchasing their tickets to ask agent for receipts or certificate. In cases where persons are unable to purchase tickets through to Galveston, they should purchase to the nearest junction point, and repurchase from that point to Galveston, taking a second certificate or receipt, covering the purchase of ticket from junctional point to Galveston, inasmuch as it will be necessary for each delegate to show certificate for the entire journey from starting point to Galveston in order to have them considered in the final count for the required number in order to secure the reduced rate.

H. G. THOMPTON, G. P. & T. A.

HOTEL RATES.

Beach Hotel, $2.50 and upward per day.
Tremont Hotel, $2.50 to $3.00 per day.
Washington Hotel, $2.50 per day.
City Hotel, $1.50 per day.

*Note.*—Private Boarding Houses $1.00 per day. Those who desire to do so can secure accommodations in advance by addressing Louis Schleisinger, Esq., 406 Tremont St., Galveston.

THE MEETING.

The meeting will be held at Harmony Hall, Corner 22nd and Church Streets, which is within a few blocks of the Union Depot, and accessible by street cars. Members are requested to report at the Hall promptly and early, in order to register as far as possible before the opening, which will be at 11.00 o'clock a. m. Members of the reception committee will be present at 8 o'clock to attend to registration and furnish badges. The latter will only be furnished after registering. New members will receive badges after formal application, and exhibiting the Treasurer's receipt.

The following is a partial list of papers. It is incomplete simply from the fact that in spite of reiterated urging upon my part and officers of sections, titles have not been forthcoming.

H. A. WEST. M. D.. Secretary,
And Chairman of Committee of Aarangements.

---

### PRELIMINARY PROGRAM.

---

SECTION ON GENERAL MEDICINE.

1. Report of the Chairman, J. C. Loggins, M. D., Ennis.
2. "The Association of Diseases and Morbid Processes," H. A. West, M. D., Galveston.
3. "The Action and Uses of Pental," David Cerna, M. D., Galveston.
4. "Metastasis in Cancer of the Stomach," Allen J. Smith, A. M., M. D., Galveston.
5. "The Continued Fevers of Texas Classified," B. F. Brittain, M. D., Baird.
6. "A Study of the Factors Concerned in the Reconversion of Peptone into Albumen, and their Relation to Certain Organic Diseases," James Kennedy, M. D., San Antonio.
7. "Opium Suicides, their Prevenfion and Treatment," Report of 15 cases; E. W. Capps, M. D., Fort Worth.
8. "Report of Ten Cases of Membranous Croup, with Remarks on Treatment," I. E. Clark, M. D., Schulenburg.
9. "Concussion of the Spine," C. H. Wilkinson, M. D., Galveston.

Clay Johnson, M. D., Secretary, Corsicana.

SECTION OF OBSTETRICS AND DISEASES OF CHILDREN.

1. Report of Chairman, Irwin Pope, M. D., Tyler, Texas.

2. "Influence in Development of Diseases," H. P. Cooke, M. D., Galveston.

3. "Paracentisis Capitis in Hydrocephalus," Report of a case; N. A. Olive, M. D.; Waco.

4. "Placenta Prævia with New Instruments for Treating Same," Q. C. Smith, M. D., Austin.

E. K. McKenzie, M. D., Secretary, Tyler.

SECTION ON SURGERY.

1. Report of Chairman, A. B. Gardner, M. D., Bellville, Tex.

2. "Whitehead's Operation for Hæmorrhoids Considered from an Anatomical and Pathological Standpoint," James E. Thompson, F. R. C. S. Eng., Galveston.

3. "Rapid Operating as a Factor in Surgical Success," A. Morgan Cartledge, M. D., Louisville, Ky.

4. "A Case of Spleenectomy with Recovery," J. F. Y. Paine, M. D., Galveston.

5. "The Treatment of Depressed Fractures of the Skull," P. C. Coleman, M. D., Colorado.

6. "Gunshot Wound of the Chest and Spine," J. E. Prince, M. D., Big Springs.

7. "The Treatment of Tetanus," Geo. H. Lee, D. M., Galveston.

8. Treatment of Perforating Typhoid Ulceration by Operative Measures," James E. Thompson, F. R. C. S. Eng., Galveston.

9. "A Modified Whitehead's Operation for Hæmorrhoids," A. F. Sampson, M. D., Galveston.

10. "Bone Excision in the Forearm," W. R. Blaylock, M. D., McGregor.

11. "Incised Wound of Abdomen—Removal of one-third of Omentum—Recovery," I. E. Clark, M. D., Schulenburg.

12. "Moist Gangrene with Amputation," J. D. Osborn, M.D., Cleburne.

M. Smith, M. D., Secretary, Black Jack Grove.

SECTION ON MEDICAL JURISPRUDENCE.

1. "Progress Made in the Treatment of the Insane," John Preston, M. D., Chairman.

2. "Hypnotism," Matthew M. Smith, B. Sc., A. M., M. D., Austin.

3. "Transverse Myelitis, with Cases," Allen J. Smith, A. M., M. D., Galveston.

B. M. Worsham, M. D,, Secretary.

SECTION ON STATE MEDICINE, ETC.

1. Report of Chairman, C. M. Rosser, M. D., Dallas.

2. "Some Thoughts on Higher Medical Education and Medical Ethics," David Cerna, M. D., Ph. D., Galveston.

J. S. Letcher, M. D., Secretary, Dallas.

SECTION ON GYNÆCCLOGY.

1. Address of Chairman, J. F. Y. Paine, M. D., Galveston.

2. "The Pathology and Treatment of Intra-Pelvic Inflammation in Women," Louis McMurtry, M. D., Louisville, Ky.

3. "A Contribution on the Pathology of the Fourchette," B. E. Hadra, M. D., Galveston.

4. "Rupture of the Perineum," B. H. Vaughn, M. D., Vaughan.

5. "Two Cases of Unilateral Oophorectomy with Practical Deductions pertaining thereto," J. Cummings, M. D., Austin.

6. "Cervical and Corporeal Endometritis," Wm. M. Cunningham, M. D., Bastrop.

7. "Preventive Gynæcology—Important Factors in the Causation of Uterine Disorders and Neurasthenia," M. M. Scott, M. D., Brownwood.

8. "Use of Electrolysis in Stenosis of the Cervix Uteri," Arthur S. Wolff, M. D., Brownsville.

9. "Pelvic Peritonitis and Cellulitis," Wm. Keiller, F. R. C. S. Ed., Galveston.

10. Paper, title not received, Joseph Price, M. D., Philadelphia, Pa.

Geo. H. Lee, M. D., Secretary, Galveston.

SECTION ON OPHTHALMOLOGY AND OTOLOGY.

1. "A Contribution to the Study of Insufficiencies of Ocular Muscles and the Measures Directed to their Relief," George P. Hall, M. D., Galveston.

2. "Trachoma, or Granular Conjunctivitis," R. E. Haughton, M. D., Midland.

3. "Paralysis of Accommodation as a Sequela," Wm. Caston, M. D., Corsicana.

4. "Neurosis of the Eye due to Stricture of the Male Urethra, with Cases," John V. Spring, M. D., San Antonio.

5. "Otitis and its Treatment," W. H. Monday, M. D., Terrell.

6. "Aural Catarrh, the Importance of Early Recognition and Treatment," Wm. H. Baldinger, M. D., Galveston.

George P. Hall, M. D., Chairman, Galveston.

Wm. H. Baldinger, M. D., Secretary, Galveston.

SECTION ON DERMATOLOGY, ETC.

1. Report of Chairman, R. W. Knox, M. D., Houston.

2. "Acne," F. E. Daniel, M. D., Austin.

3. "Circumcision," R. W. Knox, M. D., Houston.

H. P. Cooke, M. D., Secretary, Galveston.

MICROSCOPY AND PATHOLOGY.

1. "Histological Demonstrations on Malignant Tumors," Allen J. Smith, A. M., M. D., Galveston.

